# Sustained Agricultural Spraying: From Leaf Wettability to Dynamic Droplet Impact Behavior

**DOI:** 10.1002/gch2.202300007

**Published:** 2023-07-19

**Authors:** Bo Wang, Jie Wang, Cunlong Yu, Siqi Luo, Jia Peng, Ning Li, Tengda Wang, Lei Jiang, Zhichao Dong, Yilin Wang

**Affiliations:** ^1^ CAS Key Laboratory of Colloid Interface and Chemical Thermodynamics CAS Research/Education Center for Excellence in Molecular Sciences Beijing National Laboratory for Molecular Science Institute of Chemistry Chinese Academy of Sciences Beijing 100190 China; ^2^ University of Chinese Academy of Sciences Beijing 100049 China; ^3^ CAS Key Laboratory of Bio‐inspired Materials and Interfacial Sciences Technical Institute of Physics and Chemistry Chinese Academy of Sciences Beijing 100190 China

**Keywords:** agricultural spray, drop impact, leaf wettability, surface microstructure, surfactant

## Abstract

Crop production and quality safety system have the potential to nurture human health and improve environmental sustainability. Providing a growing global population with sufficient and healthy food is an immediate challenge. However, this system largely depends on the spraying of agrochemicals. Crop leaves are covered with different microstructures, exhibiting distinct hydrophilic, hydrophobic, or even superhydrophobic wetting characteristics, thus leading to various deposition difficulties of sprayed droplets. Here, the relationship between wettability and surface microstructure in different crop leaves from biological and interfacial structural perspectives is systematically demonstrated. A relational model is proposed in which complex microstructures lead to stronger leaf hydrophobicity. And adding surfactant with a faster dynamically migrating velocity and reducing droplet size can improve agrochemical precise deposition. These contribute toward highly accurate and efficient targeted applications with fewer agrochemicals use and promote sustainable models of eco‐friendly agriculture systems.

## Graphical abstract

The relationship between wettability and surface microstructure in a wide range of crop leaves from biological and interfacial structural perspectives is revealed.

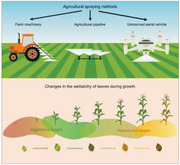

## 1 Introduction

**Figure 1:**
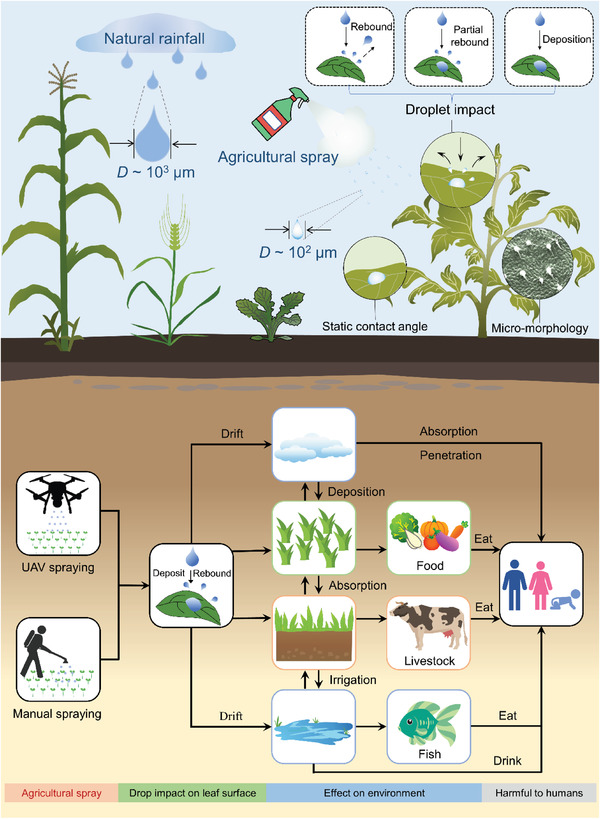
Crops are subjected to droplet impact mainly in natural rainfall and agricultural spraying, and the variations in the static wetting capacity and surface microstructure of crop leaves lead to different impact outcomes such as droplet bounce, partial bounce, and deposition. This results in inefficient spraying and pesticide contamination of the ecosystem, which enriches the human body through the food chain, posing a severe threat to human health. All graphs are hand drawings by B. Wang.

With a global crop system already under immense population pressure, food insecurity has risen to threaten up to 828 million people across the world^[^ ^1^, ^2^, ^3^ ^]^ (Figure S1, Supporting Information). Moreover, multiple unprecedented threats have further shocked global crop production and security, including COVID‐19, locust swarms, climate extremes, and violent conflicts in recent years.^[^ ^4^, ^5^, ^6^ ^]^ Pesticides are an irreplaceable and necessary guarantee for high and stable crop yields. More than 30% of global crop loss is saved by pesticides controlling weeds and defending against pests, according to the FAO.^[^ ^7^, ^8^, ^9^ ^]^ While diverse crop leaf wettability is a fundamental factor affecting the deposition of agrochemical droplets on the surface, the deposition efficiency is less than 40% because many droplets bounce and splash on the leaves or drift during the impact fall. About 90% of pesticides do not reach the target area and flow into the surrounding ecosystem,^[^ ^10^, ^11^, ^12^ ^]^ resulting in soil, water, and atmospheric pollution.^[^ ^9^, ^10^, ^13^, ^14^, ^15^ ^]^ This causes not only unnecessary financial damage to farmers but also biological enrichment^[^ ^16^, ^17^ ^]^ transferred into the human body through the food chain,^[^ ^18^, ^19^, ^20^, ^21^ ^]^ causing biomagnification, a severe threat to human health (**Figure** **1**). Therefore, a systematic understanding of surface wettability and the corresponding impact behavior is urgently needed to guide agricultural spraying and improve pesticide utilization.

Surface wettability is a fundamental property of crop leaves, which plays a significant role in plant survival.^[^ ^22^, ^23^ ^]^ From the perspective of plant physiology, the crop leaf surface has a waxy layer structure, endowing crop leaves with general hydrophobicity, which means that the leaves can inhibit evaporation while reducing the deposition of rainwater,^[^ ^24^ ^]^ and the inclined and curved morphology of leaves can also facilitate rapid drainage.^[^ ^25^ ^]^ Many plants’ leaves have evolved superhydrophobic surfaces and micro/nanostructures,^[^ ^26^, ^27^, ^28^ ^]^ especially in aquatic crops, such as rice, lotus root, taro, etc. Researchers of biomimetics and interface sciences have taken this as an essential research direction, revealing that the interaction of surface micro/nanostructures and waxy layer achieves superhydrophobicity on the leaf surface.^[^ ^26^, ^29^, ^30^ ^]^ Such extreme water repellency makes droplets hard to stay on crop leaves.^[^ ^29^ ^]^ Besides plant physiology and biomimetic research, physical studies reveal that the contact time, *t ∼* (*ρ×R* ^3^/*γ*)^1/2^, of impacting droplet on the superhydrophobic surface is at millisecond timescales, where *ρ* is the liquid density, *R* is the radius of impacting droplet, and *γ* is the surface tension. The contact time is found to not correlate with impact velocity but with droplet radius.^[^ ^31^ ^]^ A smaller radius makes a shorter contact time. Moreover, the superhydrophobic macrotexture (ridge) structure can further reduce the contact time, *t*_m_ = *t*/(2^1/2^), where *t*_m_ is the contact time under the presence of macroscopic ribs on the surface.^[^ ^32^, ^33^, ^34^ ^]^

Phytologists found that the evolved hydrophobic/super‐hydrophobic nature can benefit crop survival.^[^ ^26^ ^]^ In contrast, agronomists found that the difficulty of pesticide spray deposition on the leaf surface would weaken the advantages of human intervention and regulation of crop growth.^[^ ^35^, ^36^, ^37^ ^]^ The violent splash generated by the impact will cause the satellite droplets to scatter into the air, soil, and water.^[^ ^38^ ^]^ Even surfactant‐laden aqueous drops cannot achieve spreading on superhydrophobic leaf surfaces within such a short (millisecond timescale) impact time.^[^ ^35^, ^38^, ^39^, ^40^, ^41^ ^]^ Moreover, smaller droplet sizes are preferred in agricultural sprays for better atomization. As the droplet size decreases, the contact time is reduced,^[^ ^31^ ^]^ which makes the response to impact dynamics more complex. However, little work has been reported on the bouncing, partial bouncing, bursting, and deposition processes of tiny droplets (with a radius < 1 mm) spray on crop leaf surfaces.^[^ ^42^, ^43^ ^]^

Here, we systematically study the factors influencing crop wettability, including 55 crops covering eighteen families, and demonstrate a model for the effect of surface microstructure on leaf wettability. Moreover, the dynamic impact behaviors of droplets on varied leaves are investigated. And we modulate this behavior by a series of surfactants with various charged states and oligomeric degrees. And the reduction of droplet size also can enhance efficient deposition. The valuable results above advance understanding of the droplet impact process on crop leaves and develop methods for manipulating the droplet deposition. These understandings will provide multidisciplinary scientists, including but not limited to scholars of chemistry, physics, botany, and agronomy, with new research perspectives and research methods. The small process provided by this article gives the potential for significant scientific progress in improving agricultural sprays for a sustained and green future.

## 2 Result and Discussion

### 2.1 Crop Leaf Wettability and Surface Microstructure Morphology

To explore crop wettability, we test the contact angle on the adaxial and abaxial surfaces of a wide range of crop foliage (**Table** **1**). The crop species belong to eighteen families, including major grain crops of rice, wheat, maize, etc., major cash grain crops of soybeans, cotton, rape, beet, etc., and various vegetables and fruits.

**Table 1:** List of crop species and water static contact angle (WCA) of leaves

Species	WCA of Leaves		Location
	Adaxial	Abaxial	
*Poaceae*			
*Oryza sativa* L. (Rice)	158.7 ± 1.4	157.0 ± 1.7	Chinese Academy of Agricultural Sciences, Beijing
*Triticum aestivum* L. (Wheat)	152.4± 0.5	146.0 ± 2.9	China Agricultural University, Beijing
*Zea mays* L.(Maize)	61.7 ± 5.6	64.6 ± 5.5	China Agricultural University, Beijing
*Setaria italica* var. *germanica* (Mill.) Schred. (Millet)	140.3 ± 3.3	128.7 ± 2.8	China Agricultural University, Beijing
*Sorghum bicolor* (L.) Moench (Sorghum)	149.3 ± 3.6	133.7 ± 2.9	China Agricultural University, Beijing
*Phyllostachys edulis* (Carrière) J. Houz. (Mao bamboo)	83.7 ± 3.6	144.9 ± 2.6	China Agricultural University, Beijing
*Echinochloa crus‐galli* (L.) P. Beauv. (Barnyard grass)	150.7 ± 2.7	147.4 ± 1.1	Chinese Academy of Agricultural Sciences, Beijing
*Imperata cylindrica* (L.) P. Beauv. (Thatch)	147.7 ± 1.5	—	Haidian Park, Beijing
*Phragmites australis* (Cav.) Trin. ex Steud. (Reeds)	148.7 ± 0.9	—	Haidian Park, Beijing
*Setaria viridis* (L.) P. Beauv. (Foxtail)	146.0 ± 2.7	—	Haidian Park, Beijing
*Zingiberaceae*			
*Alpinia officinarum* Hance (Gao Liang Jiang)	94.0 ± 2.0	116.9 ± 0.7	Haidian Park, Beijing
*Brassicaceae*			
*Brassica oleracea* var. *albiflora* Kuntze (White Flowering kale)	147.9 ± 1.8	137.8 ± 2.7	Pingren Farm, Changping District, Beijing
*Brassica oleracea* var. *capitata* L. (Kale)	149.1 ± 0.6	136.4 ± 5.1	Pingren Farm, Changping District, Beijing
*Brassica oleracea* var. *botrytis* L. (Cauliflower)	140.3 ± 0.2	138.6 ± 2.7	Pingren Farm, Changping District, Beijing
*Brassica oleracea* var. *italica* Plenck (Broccoli)	146.5 ± 2.7	146.7 ± 0.7	Pingren Farm, Changping District, Beijing
*Brassica campestris* var. *purpuraria* L.H.Bariley (Purple cai‐tai)	123.3 ± 4.0	133.7 ± 2.0	Pingren Farm, Changping District, Beijing
*Brassica rapa* var. *glabra* Regel (Cabbage)	96.9 ± 4.2	105.2 ± 5.8	Pingren Farm, Changping District, Beijing
*Brassica napus* L. (Oilseed rape)	42.7 ± 0.7	64.8 ± 8.5	Pingren Farm, Changping District, Beijing
*Raphanus sativus* L. (Radish)	92.3 ± 1.3	78.2 ± 6.4	Yichun, Jiangxi Province
*Brassica rapa* var. *oleifera* DC. (Rape)	136.7 ± 3.2	140.7 ± 3.6	Yichun, Jiangxi Province
*Brassica rapa* var. *chinensis* (L.) Kitam. (Qing cai)	91.0 ± 3.6	88.0 ± 1.9	Pingren Farm, Changping District, Beijing
*Brassica juncea* (L.) Czern. (leaf mustard)	65.3 ± 3.1	99.5 ± 7.2	Pingren Farm, Changping District, Beijing
*Brassica oleracea* var. *acephala* DC. (Bare cole)	133.8 ± 4.8	140.3 ± 5.0	Shunyi Farm, Beijing
*Bromeliaceae*			
*Ananas comosus* (L.) Merr. (Pineapple)	74.8 ± 2.9	128.5 ± 3.1	Shunyi Farm, Beijing
*Solanaceae*			
*Solanum lycopersicum* L. (Tomato)	113.3 ± 4.7	125.5 ± 0.5	China Agricultural University, Beijing
*Solanum melongena* L. (Eggplant)	94.3 ± 5.1	135.5 ± 1.0	China Agricultural University, Beijing
*Capsicum annuum* L. (Pepper)	92.4 ± 4.7	91.7 ± 5.7	China Agricultural University, Beijing
*Meliaceae*			
*Toona sinensis* (Juss.) Roem. (Toon)	118.3 ± 5.5	98.1 ± 0.9	China Agricultural University, Beijing
*Lamiaceae*			
*Mentha canadensis* L. (Mint)	88.6 ± 3.1	95.5 ± 2.5	Kunming, Yunnan Province
*Perilla frutescens* (L.) Britton (Perilla)	25.0 ± 0.5	78.5 ± 2.5	Shunyi Farm, Beijing
*Asteraceae*			
*Lactuca sativa* var. *ramosa* Hort. (Ye yong Lettuce)	66.6 ± 5.4	61.3 ± 0.9	China Agricultural University, Beijing
*Lactuca sativa* L. (Lettuce)	85.6 ± 2.8	92.3 ± 3.3	China Agricultural University, Beijing
*Helianthus annuus* L. (Sunflower)	84.6 ± 4.5	79.0 ± 6.9	China Agricultural University, Beijing
*Glebionis coronaria* (L.) Cass. ex Spach (Tarragon)	69.0 ± 2.8	56.8 ± 4.2	Shunyi Farm, Beijing
*Cichorium endivia* L. (Endive)	80.7 ± 3.1	38.0 ± 6.2	Shunyi Farm, Beijing
*Lactuca sativa* var *longifoliaf*. Lam (Romaine lettuce)	80.6 ± 2.8	64.8 ± 3.4	Shunyi Farm, Beijing
*Rosaceae*			
*Fragaria × ananassa (Weston)* Duchesne ex Rozier (Strawberry)	138.2 ± 3.7	137.9 ± 2.3	Pingren Farm, Changping District, Beijing
*Cucurbitaceae*			
*Cucumis sativus* L. (Cucumber)	73.3 ± 6.7	88.9 ± 3.6	China Agricultural University, Beijing
*Momordica charantia* L. (Bitter gourd)	87.5 ± 4.9	87.9 ± 2.5	China Agricultural University, Beijing
*Luffa aegyptiaca* Mill. (Loofah)	96.8 ± 8.0	87.2 ± 1.8	China Agricultural University, Beijing
*Cucurbita moschata* (Duchesne ex Lam.) Duchesne ex Poir. (Pumpkin)	57.4 ± 3.3	73.4 ± 0.4	China Agricultural University, Beijing
*Begoniaceae*			
*Begonia fimbristipula* Hance (Purple‐backed Anemone)	79.3 ± 5.6	80.7 ± 9.8	Pingren Farm, Changping District, Beijing
*Fabaceae*			
*Glycine max* (L.) Merr. (Soybean)	86.0 ± 3.0	119.7 ± 5.9	China Agricultural University, Beijing
*Vigna unguiculata* (L.) Walp. (Cowpea)	84.7 ± 5.0	63.8 ± 1.0	China Agricultural University, Beijing
*Apiaceae*			
*Petroselinum crispum* (Mill.) Fuss (Parsley)	70.2 ± 4.4	62.0 ± 5.7	Pingren Farm, Changping District, Beijing
*Foeniculum vulgare* Mill. (Fennel)	60.9 ± 4.1	75.6 ± 2.8	Shunyi Farm, Beijing
*Amaranthaceae*			
*Spinacia oleracea* L. (Spinach)	83.7 ± 2.0	96.7 ± 3.0	Pingren Farm, Changping District, Beijing
*Beta vulgaris* L. (Beet)	67.4 ± 1.0	91.9 ± 4.3	Pingren Farm, Changping District, Beijing
*Malvaceae*			
*Gossypium hirsutum* L. (Cotton)	48.4 ± 0.3	33.3 ± 0.6	China Agricultural University, Beijing
*Abelmoschus esculentus* (L.) Moench (Abelmoschus esculentus)	104.7 ± 0.2	98.0 ± 4.2	Pingren Farm, Changping District, Beijing
*Convolvulaceae*			
*Ipomoea batatas* (L.) Lam. (Sweet potato)	58.5 ± 5.3	80.5 ± 7.8	China Agricultural University, Beijing
*Ipomoea aquatica* Forssk. in Forssk. & Niebuhr (Water spinach)	61.7 ± 5.6	133.8 ± 2.7	Ningyang, Tai'an, Shandong Province
*Amaryllidaceae*			
*Allium tuberosum* Rottler ex Spreng. (Jiu cai)	116.8 ± 5.2	133.8 ± 2.7	Yichun, Jiangxi Province
*Allium fistulosum* L. (Welsh onion)	49.4 ± 5.5	124.3 ± 1.3	Shunyi Farm, Beijing
*Moraceae*			
*Ficus carica* L. (Fig)	22.2 ± 3.2	95.3 ± 5.6	China Agricultural University, Beijing

The crop wettability can be broadly classified into four categories: hydrophilic, 0° to 90° (I), weak hydrophobic, 90° to 120° (II), strong hydrophobic, 120° to 150° (III), superhydrophobic, 150° to 180° (IV). The distinction between hydrophilic and hydrophobic can be defined according to Young's equation.^[^ ^44^ ^]^ From the mathematical equation, the upper limit for defining the hydrophilic surface is 90°. Considering surface roughness, 65° is found as the dividing boundary between hydrophilic and hydrophobic surfaces.^[^ ^45^ ^]^ In addition, for the hydrophobic surface, 120° is used to divide the weak hydrophobic surface and the strong hydrophobic surface in this work, which is based on the maximum contact angle of the smooth material surface of Teflon. Furthermore, the superhydrophobic surface is divided into high adhesion surface and low adhesion surface, which can be determined according to the Wenzel equation,^[^ ^46^ ^]^ cos*θ*´ = *r*·cos*θ*, and the Cassie‐Baxter equation,^[^ ^47^ ^]^ cos*θ*´ = *f*·cos*θ*·(1‐ *f*), where *θ*´ is the apparent contact angle on a rough surface, *θ* is the intrinsic contact angle on a smooth surface, *r* is the surface roughness, f is the fraction of the solid/water interface, and (1 – *f*) is the fraction of the air/water interface. Increasing the leaves’ surface roughness can reinforce the inherent hydrophobic or superhydrophobic properties.^[^ ^30^ ^]^

**Figure 2:**
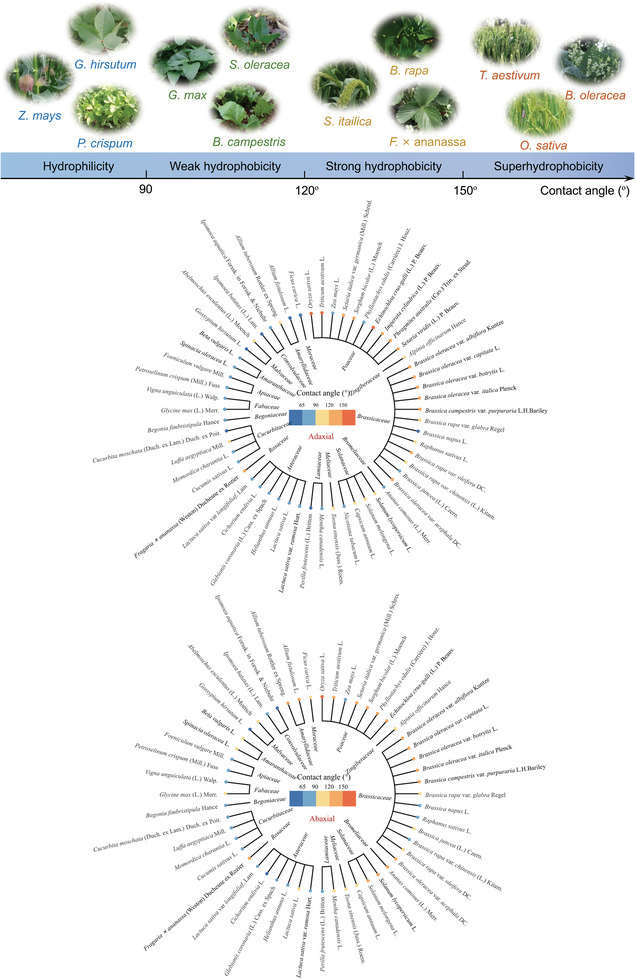
Delineation of the boundaries of crop wettability and clustering analysis overview of wettability from crop adaxial and abaxial surface by plant Taxonomy, respectively. Data for the adaxial contact angle of tobacco (*Nicotiana tabacum* L.) leaves are derived from ref.[48]

**Figure** **2** illustrates the contact angles of the adaxial and abaxial surfaces of 55 kinds of crop leaves. Cluster analysis classifies these 55 species into 18 family groups, including *Poaceae*, *Brassicaceae*, *Solanaceae*, *Asteraceae*, *Cucurbitaceae*, *Fabaceae*, *etc*., and reveals that the crops from the same family mostly show similar leaf wettability by plant taxonomic comparisons: the leaves of *Poaceae* crops generally show a strong hydrophobic or superhydrophobic state except maize, the leaves for most of the *Brassicaceae* and *Solanaceae* crops are in the hydrophobic state, and the leaves of *Asteraceae* and *Cucurbitaceae* crops are in the hydrophilic state. Although the number of crop samples from other families is relatively insufficient, the same pattern can be reflected. This implies that leaves from the same crop family usually have similar surface waxy layer composition and surface microstructure.

**Figure 3:**
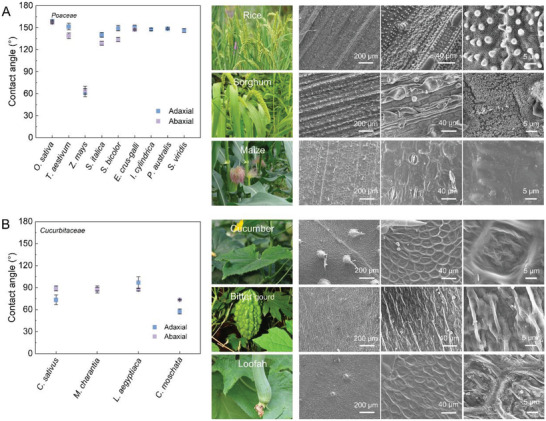
The range of contact angles for *Poaceae* and *Cucurbitaceae* crops and corresponding images of leaf micromorphology. A) Contact angles of *Poaceae* crops and SEM images of surface microstructures of typical *Poaceae* crops, rice, sorghum, and maize. B) Contact angles of *Cucurbitaceae* crops and SEM images of surface microstructures morphology of typical *Cucurbitaceae* crops, cucumber, bitter gourd, and loofah.

Next, we find that the crops of the same family have similar water contact angles and microstructures on their leaves (Figure 3A,B and Figures S2 and S3, Supporting Information). For the examples of the *Poaceae* crops, the leaf contact angles are primarily between 130° and 160°. The surface microstructures all show typical anisotropic periodic arrays of microcapillaries covered with dense needle‐like structures. Unlike other crops of the *Poaceae* family, maize has a much larger leaf size and specific traits that lead to a low density of surface microstructures, therefore exhibiting a lower contact angle. For the *Cucurbitaceae* crop, the surface microstructures of leaves all show a distinct isotropic periodic ring arrangement, where the waxy layer is smoother and has no dense needle‐like structures (**Figure** **3**B; Figures S2 and S3, Supporting Information). As a result, the contact angles on the *Cucurbitaceae* leaves are mostly in the range of 60–90°. In general, crop leaves wettability and surface microstructure are relatively similar in the same family. This diversity decline helps to better explain the purposefulness of the crop domestication process by human selection.

Due to the evolutionary characteristics of the crops for survival and physiological functions, there are differences in the contact angles presented by the adaxial (facing the sun) and abaxial (near the ground) surfaces of crop leaves. To reduce transpiration, prevent water loss, and improve photosynthesis,^[^ ^49^, ^50^ ^]^ the adaxial surface has a thicker and more complex waxy layer,^[^ ^50^ ^]^ leading to a higher contact angle and a more hydrophobic or superhydrophobic surface (Table 1). The function of the abaxial surface is to complete the gas exchange between the leaf and the external environment, therefore the abaxial surface has more stomata^[^ ^51^ ^]^ and a thinner wax layer, resulting in a lower contact angle compared to the adaxial surface. This feature is not only in *Poaceae* and  *Cucurbitaceae* crops but also in most of the leaves of the other families we observed.

**Figure 4:**
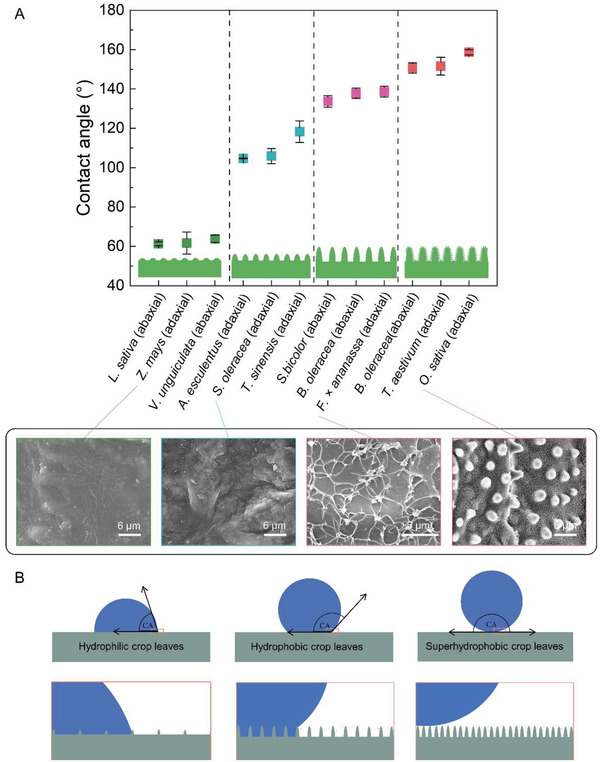
The relationship between surface microstructure and leaf wettability. A) Contact angles of crop leave with various surface microstructures. B) Models of static contact angles with different surface microstructures.

**Figure 5:**
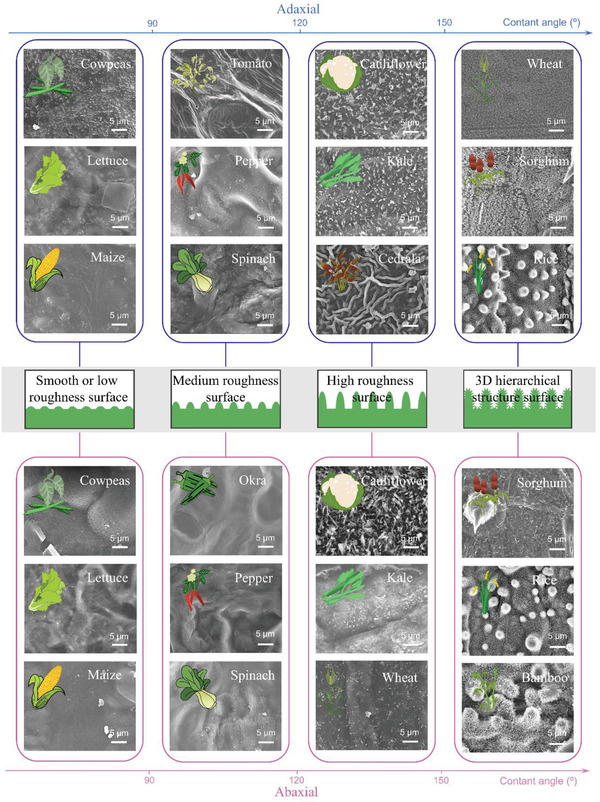
Correspondence between the roughness of the leaf surface microstructure and the magnitude of the contact angle demonstrates the model's accuracy in Figure 4A,B.

**Figure** **4**A summarizes the relationship between crop leaf wettability and surface microstructure. The leaves with higher roughness and complexity of surface microstructure exhibit higher contact angles and more hydrophobicity/superhydrophobicity: I) The leaf surfaces with a smooth or low cuticle fold structure, such as lettuce, maize, cowpea, etc., show low contact angles between 60° and 90°; II) The leaf surfaces with medium cuticle fold structure or microstructures of general complexity and array density, such as spinach, toon, coffee wasabi, etc., show contact angles between 90° and 120°; III) The leaf surfaces with high cuticle fold structure or microstructures of high complexity and array density, such as cauliflower, white flowering kale, sorghum, *etc*., show contact angles between 120° and 150°; IV) The leaf surfaces with 3D hierarchical multi‐layered dense arrays of arranged microstructures, such as rice, wheat, barnyard grass, etc., show contact angles above 150°, belonging to the category of the superhydrophobic surface. These results lead us to propose a universal and simple structure‐wettability model, where surface roughness plays a crucial role in the case of similar intrinsic contact angles of the waxy layer of crop leaves (Figure 4B). When the surface roughness is high enough, crop leaves are of a superhydrophobic surface, thus there is a large amount of air in the protrusions and depressions of the rough surface, and the droplets exist only on the above of the microstructure, which is the Cassie state,^[^ ^47^ ^]^ apparent contact angle, *cos* θ_f_ = *f*_s_ (*L*/*l*)^D − 2^ *cos*θ − *f*_v_, where *θ*_f_ is the contact angle on the rough surface, *θ* is the intrinsic contact angle, (*L*/*l*)^D‐2^ is the surface roughness factor, *L* and *l* are separately the upper and lower limit scales of the fractal behavior of the surface, *D* is the fractal dimension, and *f_s_* and *f*_v_ are the fractions of the surface under the water droplet occupied by solid material and air, respectively, and (*f*_s_ + *f*_v_ = 1). Since *θ*_f_ >> *θ*, the droplets slide easily. When the droplet can fill the grooves on the rough surface and form a fully wetted state, the crop leaf is of a hydrophilic surface in the Wenzel state,^[^ ^46^ ^]^ cos*θ*_f_ = *r*·cos*θ*, *θ ˂ θ*_f_ *˂* 90°, where the contact angle between the droplet and the surface is relatively low and not easy to slide. While a crop leaf is hydrophobic, the droplets will partially enter the gaps of the raised structure, which is the intermediate state between the Cassie state and the Wenzel state, i.e., the droplet is partially pinned, and the remaining part of the droplet stays on the surface. At this point, *θ*_f_ > 90°. Furthermore, we confirm the above model for the relationship between the surface micromorphology and wettability of crop leaves by observing the surface microstructure morphology of a variety of crops (**Figure** **5**).

### 2.2 Crop Leaf Wettability and Droplet Impact Behavior

**Figure 6:**
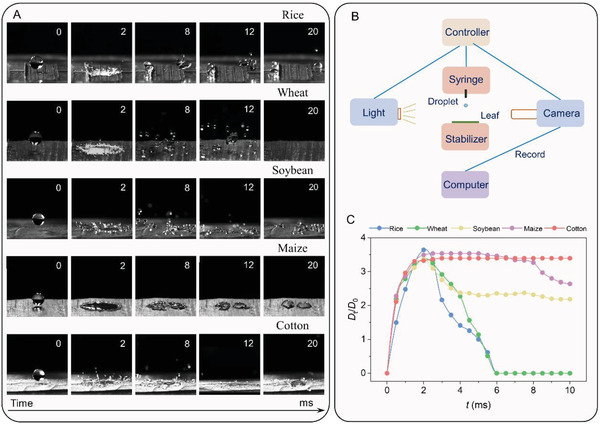
Droplet impact behavior on crop leaves with different wettability. A) Images of the impact process of pure water droplets. B) Logic diagram of the droplet impact experiment. C) Variation of droplet deposition factor as a function of time.

The deposition efficiency of impacting droplets should be associated with leaf wettability and droplet characteristics, but this remains to be explored. Based on the above measurements about the leaf wettability and microstructures, several crop leaves with typical wetting characteristics are selected to present representative deposition results by pure water droplet impact: droplets bounce ultimately on the superhydrophobic rice and wheat leaf surfaces, partially on the hydrophobic soybean leaf surface and broken into numerous small satellite droplets, deposit on the hydrophilic maize leaf surface, and deposit and spread on the more hydrophilic cotton leaf surface (**Figure** **6**A). Thus, we identify that well‐wetted crop leaves maintain better deposition efficiency.

Meanwhile, contact angle hysteresis is an essential indicator of reflecting droplet wettability, following the classification of leaf wettability as mentioned above. Here we select several typical crops and illustrate them with droplet impact results (Table S1 and Figure S5, Supporting Information). We find that lower contact angles of crop leaves generally have a greater corresponding contact angle hysteresis. Droplets are easier to deposit on the surface after impacting the leaf.

Next, the time‐resolved evolution of the normalized deposition diameter factor (*D*_t_/*D*_0_) for the impacting droplet was studied. The ratio of the droplet deposition diameter on the leaf surface to the initial diameter of the droplet is the normalized deposition factor of the droplet, *D*_t_/*D*_0_. The value of the normalized deposition factor represents the deposition capacity of the droplet on the impacting surface, with a larger value representing a larger deposition area of the droplet on the solid surface; a value of zero means that the droplet bounces off the surface. For millimeter droplets (*D*_0_ ≈ 2 mm), all the maximum values of *D*_t_/*D*_0_ are all ≈3.5, and all of them reach their maximum at ≈ 2.0 ms (Figure 6C), which is highly consistent with the previously obtained conclusion.^[^ ^31^ ^]^ The maximum deposition diameter of the droplets after impact and the corresponding contact time is only related to the droplet radius but do not depend on the other factors at a given velocity. More efficient uniform deposition can be observed in well‐wetted crop leaves, maintaining higher *D*_t_/*D*_0_ after stabilization.

**Figure 7:**
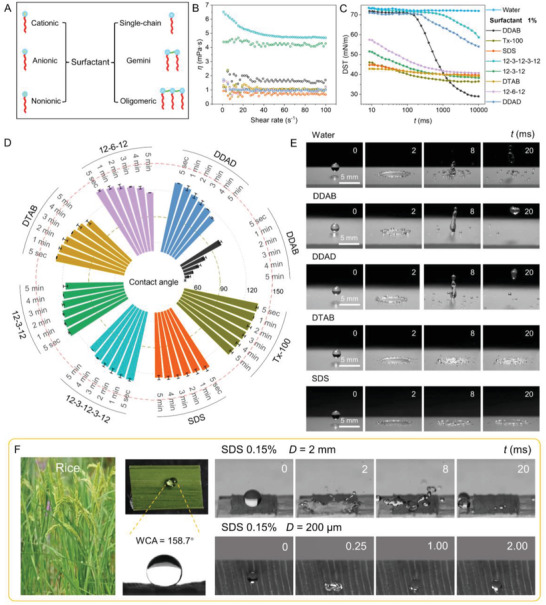
Effects of surfactants on impact behavior. A) Classification of surfactants. B) Shear viscosity curves of different surfactants with a concentration of 1%. C) The dynamic surface tension (DST) of different surfactants as a function of time. D) Static contact angles of surfactant‐laden droplets on superhydrophobic surfaces at different time scales. E) The process of droplets impacting a superhydrophobic surface with different surfactants. F) Optical picture of rice crop and the process of droplets with various diameters impacting superhydrophobic rice leaves.

The surface activity and aggregation behavior of surfactants are accomplished by the synergy of multiple weak intermolecular interactions, and here we have investigated the physical properties and the impacting behavior of water droplets with a variety of surfactants (Figure S5, Supporting Information) on superhydrophobic surfaces (**Figure** **7**A). As reported for the polymer‐laden droplets, higher shear viscosity consumes more kinetic energy during the spreading and retraction stages of droplet impact, correspondingly decreasing the phase line mobility, and thus inhibiting droplet rebound. However, herein the solution shear viscosity of most surfactants (Figure 7B) is close to that of water, indicating that shear viscosity is not expected to be a controlling factor. In contrast, the solutions contain the linear cationic quaternary amine salt trimeric surfactant (12‐3‐12‐3‐12) and the cationic gemini surfactants 1,3‐bis(dodecyl dimethyl)‐brominated diamine (12‐3‐12), have higher viscosity as medium viscosity fluids, which increase the viscoelasticity of the solutions owing to the easier formation of spherical or worm‐like micelles.^[^ ^52^, ^53^, ^54^, ^55^ ^]^

Dynamic surface tension has been considered an essential factor affecting droplet deposition during the impact process. The magnitude of dynamic surface tension is a good indicator of the migration rate of surfactant molecules. Thus, the time evolution of the dynamic surface tension for the surfactants (Figure 7C) is measured. The initial surface tension values in the measured interval of the single‐chain surfactants, sodium dodecyl sulfate (SDS) and dodecyl trimethyl ammonium bromide (DTAB), are both low, and the whole process does not exhibit a clear induction period, indicating that the molecules have a relatively fast migration rate. As the length and number of hydrophobic chains increase, such as didodecyl dimethyl ammonium bromide (DDAB), cationic stellate oligomeric surfactant tris(1‐carbonyl, 3‐dodecyl‐dimethyl quaternary ammonium) tris(2‐aminoethyl) amine trichloride (DDAD), 12‐3‐12‐3‐12, the surface tension values are all around 72 mN/m at the initial stage of the measurement, which is the same as that of pure water. Although the tension curve has a significant induction period, it only produces a significant decrease 200 ms and does not yet reach equilibrium even at 10,000 ms. This demonstrates a relatively slow molecular diffusion rate due to the large molecular weight of the oligomeric surfactants and double hydrophobic chain surfactants.

Figure 7D demonstrates the evolution of the static contact angle of the surfactant droplets with time. In contrast to pure water droplets, the droplet contact angle decreases significantly with the addition of surfactants. Moreover, for most surfactants, the static contact angle decreases slightly but remains stable for a few minutes. The exception is that the contact angle of droplets containing DDAB on the superhydrophobic surface decreases remarkably with time, indicating that DDAB has an excellent static wetting ability. This phenomenon is associated with its aggregation morphology as vesicles in solution.

Next, the impact behavior of surfactant droplets is compared (Figure 7E), and the droplets containing DDAB or DDAD exhibit a bounce behavior resembling that of the pure water droplet. In contrast, SDS and DTAB show promising deposition. This is inconsistent with the static contact angle. The static wetting process cannot match the time scale of the impact behavior on account of the transient nature of the impact process but is more dependent on the dynamic surface tension within the impact time domain, i.e., a faster migration rate of the surface activator molecules is beneficial for droplet deposition. This is consistent with the dynamic surface tension curves. The droplet formation time from extrusion to impact is ≈245 ms (available in Experimental Section), and the surface tension value for this moment is 40.6 mN/m (DTAB), 40.8 mN/m (SDS), 64.5 mN/m (DDAB), 70.1 mN/m (DDAD), respectively.

To further guide agricultural spraying applications, “small size” droplet impact behavior is studied in detail. The “small size” (drop diameter is ≈200 µm) is more consistent with the droplet diameter in actual applications, and the droplets with very low concentrations of surfactants can deposit on extremely superhydrophobic rice leaves without sputtering and satellite droplet generation as opposed to millimeter‐sized droplets. The larger specific surface area and the lower initial kinetic energy facilitate the adhesion of the impact droplets on the blade and prevent the formulation of a liquid film morphology with large deformations, which also reduces the Kelvin–Helmholtz instability, *k*_max_ ∼ 2*ρ*_a_·*U* ^2^/3*γ*, where *ρ*_a_ is the air density, *U* is the relative velocity between the gas and liquid, and *γ* is the fluid surface tension. The decrease of *k*_max_ suggests that the impact of drops driven by the inertial force and the capillary force on surfaces causes nonuniform distribution and satellite droplet splashing of the drops. The large specific surface area also enables faster migration of surfactant molecules from the bulk phase to the interface, making the droplet more “sensitive” to incorporating surfactant.

## 3 Conclusion

**Figure 8:**
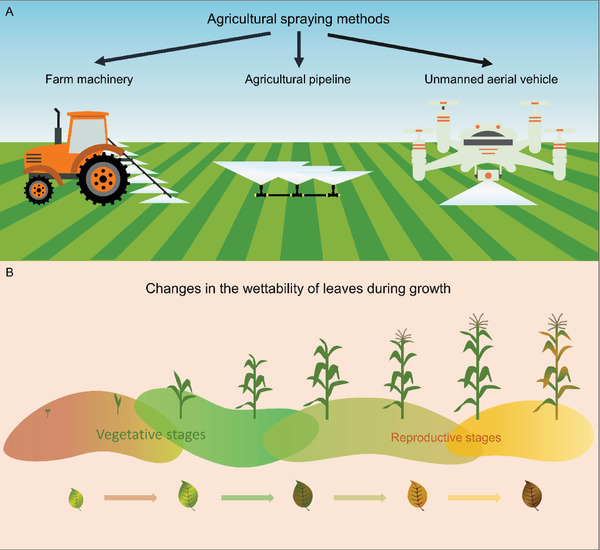
Multiple methods of agricultural spraying and extensive crop leaf wettability characteristics. A) Several typical agricultural spraying methods. (B) Variation of leaf wettability with crop growth stages.

In conclusion, these “bottom‐up” approaches by modulating surfactants and adjusting droplet size become more potent for improving spraying efficiency when integrated with “top‐down” traits by leaf wettability. Diverse crop leaf wettability dictates the importance of modifying the characteristics of the spray droplets (appropriate surfactant addition, droplet size adjustment) according to specific conditions rather than applying them mechanically. The long‐term development of agricultural technology led to various spraying methods, like manual spraying, agricultural sprinkler truck spraying, sprinkler irrigation spraying, unmanned aerial vehicle (UAV) spraying, etc. (**Figure** **8**A). Particularly, by using UAVs to control the height of spray in the field, researchers can acquire extensive deposition data for efficient and comprehensive classification. This method makes adjusting the droplet size and spraying speed much easier. Growth stages also confer variability in leaf microstructure^[^ ^56^ ^]^ (Figure 8B and Figure S6, Supporting Information), making efficient spraying much more complicated. In our vision for the future, it will be essential to collect further data on crop leaf nature and the capacity of surfactants to improve droplet deposition to resolve the limitations of spray efficiency. For hydrophobic and even superhydrophobic crops such as rice, wheat, and sorghum, reducing the droplet size and using the surfactant additive with a high molecular migration rate can improve efficient deposition. But for more hydrophilic crops such as maize and soybean, selecting larger droplets ensures deposition while also reducing droplet drift contamination. Understanding the leaf wettability and associated impact behavior provides a valuable window into sustainable agricultural production and gives essential insights vital to address the ongoing demand for improved crop yield and quality.

## 4 Experimental Section

### Chemicals

Polyethylene glycol *tert*‐octyl phenyl ether (TX‐100), sodium dodecyl sulfate (SDS), dodecyl trimethyl ammonium bromide (DTAB), and didodecyl dimethyl ammonium bromide (DDAB) were purchased from Sigam‐Alfa, and the purity was higher than 99.9%. Cationic Gemini surfactants 1,3‐bis(dodecyl dimethyl)brominated diamine (12‐3‐12), 1,6‐di(dodecyl dimethyl)diammonium bromide (12‐6‐12), linear cationic quaternary amine salt trimeric surfactant 12‐3‐12‐3‐12, and cationic stellate oligomeric surfactant tris(1‐carbonyl, 3‐dodecyl dimethyl quaternary ammonium) tris(2‐aminoethyl) amine trichloride (DDAD), which were synthesized and purified according to the corresponding papers.^[^ ^55^, ^57^, ^58^, ^59^ ^]^ Deionized water (18.2 MΩ cm) from Milli‐Q equipment was used in all experiments.

### Collection of Crop Leaves

Crops leaves were provided from the China Agricultural University, Chinese Academy of Agricultural Sciences, Beijing Haidian Park, Shunyi Farm from Beijing, Pingren Farm from Beijing, and some farms in Ningyang, Shandong, Kunming, Yunnan, and Yichun, Jiangxi. Field collections spanning 24°N (Kunming) to 41°N (Beijing) in five locations. All crop leaves were collected in the flowering stage and stored clean and dry at 4 °C. Three leaves of each species were collected. All experiments were completed within 36 h after collection, the prioritizing of experiments was the contact angle measurement, the impact test, and the SEM leaf surface micromorphology observation. The contact angle and droplet impact tests were completed within 12 h of collection. Five positions were selected for data reading.

### Surface Wettability Measurement

The contact angle (CA) of crop leaves was measured using a contact angle measurement device (DSA25s, KRUSS, Germany). All contact angle measurements were carried out five times. The droplet diameter was controlled to 2 mm. The experimental environment for contact angle measurements was maintained at 25 °C and 25%RH.

The measurements were performed using the needle‐in‐drop sessile drop. The CA was measured by slowly pumping water in and out of a needle using a motorized syringe, which was close to the sample, the tip of the needle was embedded in the water drop. Water was pumped to the drop slowly from the syringe via the needle, and the droplet contacted the surface, and the needle gradually detached from the droplet.^[^ ^60^, ^61^ ^]^

The curved leaves were cut to appropriate dimensions, stuck flat onto the glass plate, and then measured. The obtained data are shown in Table 1.

### Microscopic Morphological Observation of Crop Leaves

The surface microstructure images of crop leaves were obtained by a field‐emission Scanning Electron Microscope (SEM) at 10 kV (Hitachi S‐4800, Japan).

Fresh and clean crop leaves were selected, cut to a suitable size, fixed to the SEM sample stage using fluid conductive silver adhesive, and put into the ion sputterer for gold spraying. The vacuum was controlled at 10^−4^ kPa or less, and the spraying time was at 180 s.

### Droplet Impact Experiment

The dynamics of droplets impacting the crop leave surfaces were captured from a side view by using a high‐speed camera, shoot high‐speed camera (FASTCAM Mini UX100 Photron Japan) at a frame rate of 2000 fps with a shutter speed of 1/20 000 s for the millimeter droplet impact experiment, and shoot high‐speed camera (Photron FASTCAM NOVA S9, Japan) at a frame rate of 8000 fps with a shutter speed of 1/80 000 s for hundreds‐micron‐level droplet impact. The experimental light source was provided by a high‐brightness white 80 W LED lamp (Shanghai JQJY high‐tech Co. LTD, China). Data reliability was guaranteed by repeating the experiment at least three times, performed at 25 °C and 25%RH. The method and the concrete experimental system of the droplet impact experiment are shown in Figure 6B and Figure S4 (Supporting Information).

The leaf was put on the horizontal stabilizer, and the droplet was squeezed out through the syringe to impact the blade surface. The needle type was 28G, and the droplet extrusion rate was 0.2 µL s^−1^. In this method, the initial velocity of the droplet was nearly zero, the droplet diameter was controlled to 2 mm. And the fall height determined the rate of impact. The fall height was controlled as 30 cm, thus the impact velocity of drop, *v* = (2*g*·*h*)^1/2^ = 2.45 m s^−1^, where *h* is the fall height, *g* is the gravitational acceleration, and *v* is the droplet impact velocity. Meanwhile, a high‐speed digital camera was used to film the movement of the droplet before impact. The impact velocity of the droplet was calculated from a distance between two adjacent frames to be 2.42 m s^−1^, which was close to the rate calculated for a droplet in free fall, assuming no air resistance.^[^ ^62^ ^]^ The diameter of the droplet was also further confirmed by filming as 2 mm.

### Shear Viscosity Measurement

The shear viscosity of surfactant solutions was measured by the advanced rotational rheometer (MCR302, Anton Paar, Austria), with a shear rate range of 0.05–500 s^−1^. And the solution concentration was 1%.

### Fabrication of Superhydrophobic Surface

The polymer‐particle dispensed solution was prepared by adding 1.0 mL of Capstone ST‐200 (DuPont Co.) solution and 1.0 g of hydrophobic fumed silica nanoparticles (average particle size of 14 nm; Evonik Degussa Co.) in 5.0 mL of acetone and 20.0 mL of ethanol. The solution was mixed and stirred for 30 min in a closed bottle. The commercial glass plates were first cleaned with acetone, ethanol, and deionized water, and then were dipped in the solution at a speed of 80 mm s^−1^ and pulled out from the solution at 100 mm s^−1^. Due to the solvent's rapid evaporation, the semitransparent membrane quickly transformed into a white coating with high water repellency. And CA of the superhydrophobic surface was 160.3 ± 1.9^o^.

### Dynamic Surface Tension Test

The maximum bubble pressure method determines the dynamic surface tension of different surfactant solutions, measured using the dynamic surface tension instrument (Kruss BP100, Germany). The diameter of the capillary used to generate the bubbles was 0.215 mm. And the measured surface age was from 7 to 10 000 ms, with the experimental temperature at 25.0 °C.

## Conflict of Interest

The authors declare no conflict of interest.

## Author Contributions

B.W., Z.D., and Y.W. designed the research; B.W. performed the experiments with the assistance from J.W., C.Y., S.L., J.P., N.L., and T.W.; B.W., Z.D., and Y.W. analyzed the data; B.W. wrote the original manuscript and Z.D. and Y.W. revised it; Z.D., Y.W., and L.J. supervised the research. All authors discussed the results and commented on the manuscript.

## Supporting information

Supporting Information

## Data Availability

The data that support the findings of this study are available in the supplementary material of this article.
