# Supplementary material for: Sustained Agricultural Spraying: From Leaf Wettability to Dynamic Droplet Impact Behavior
Source: Glob Chall. 2023 Jul 19;7(9):2300007. doi: 10.1002/gch2.202300007 (PMC10517293; doi:10.1002/gch2.202300007)
Supplement: Supplementary file 1 — Supporting Information [file GCH2-7-2300007-s001.pdf]

Global Challenges
Open Access

Supporting Information

for *Global Challenges.*, DOI 10.1002/gch2.202300007

Sustained Agricultural Spraying: From Leaf Wettability to Dynamic Droplet Impact Behavior

Bo Wang, Jie Wang, Cunlong Yu, Siqi Luo, Jia Peng, Ning Li, Tengda Wang, Lei Jiang, Zhichao Dong and Yilin Wang**

Supplementary figures

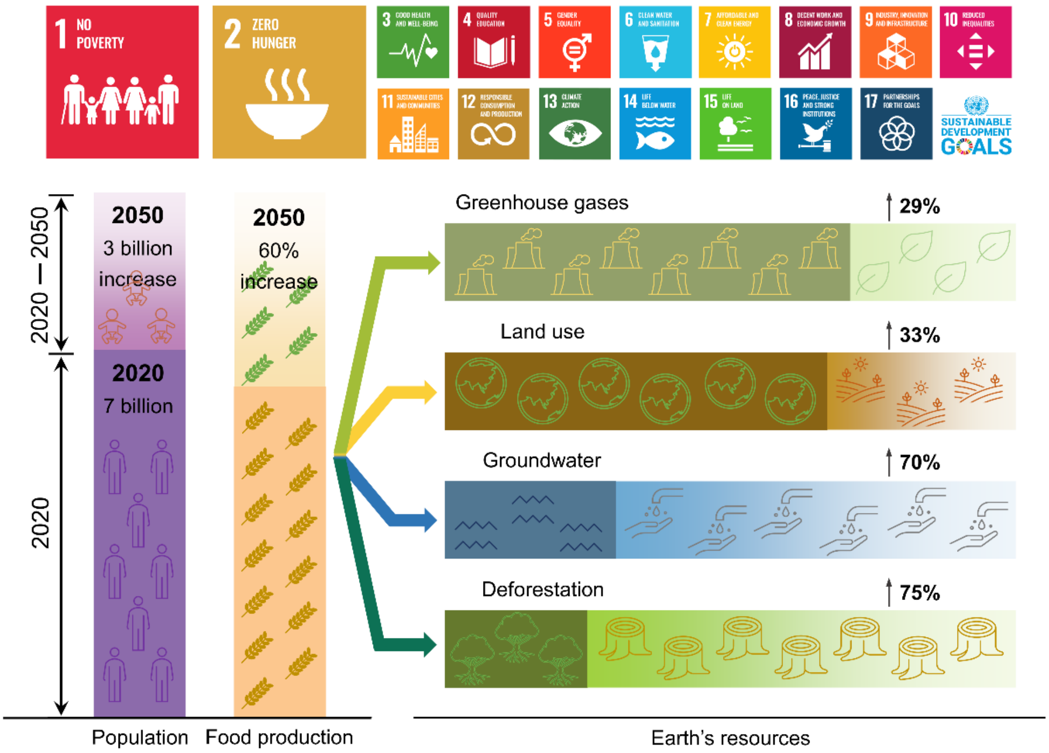

Figure S1. Population growth puts more pressure on food production, which requires higher crop yields. More frequent agricultural activities result in greater resource consumption in terms of greenhouse gas emissions, land resources, groundwater resources, and forest resources. Data are derived from **Reference 3**.

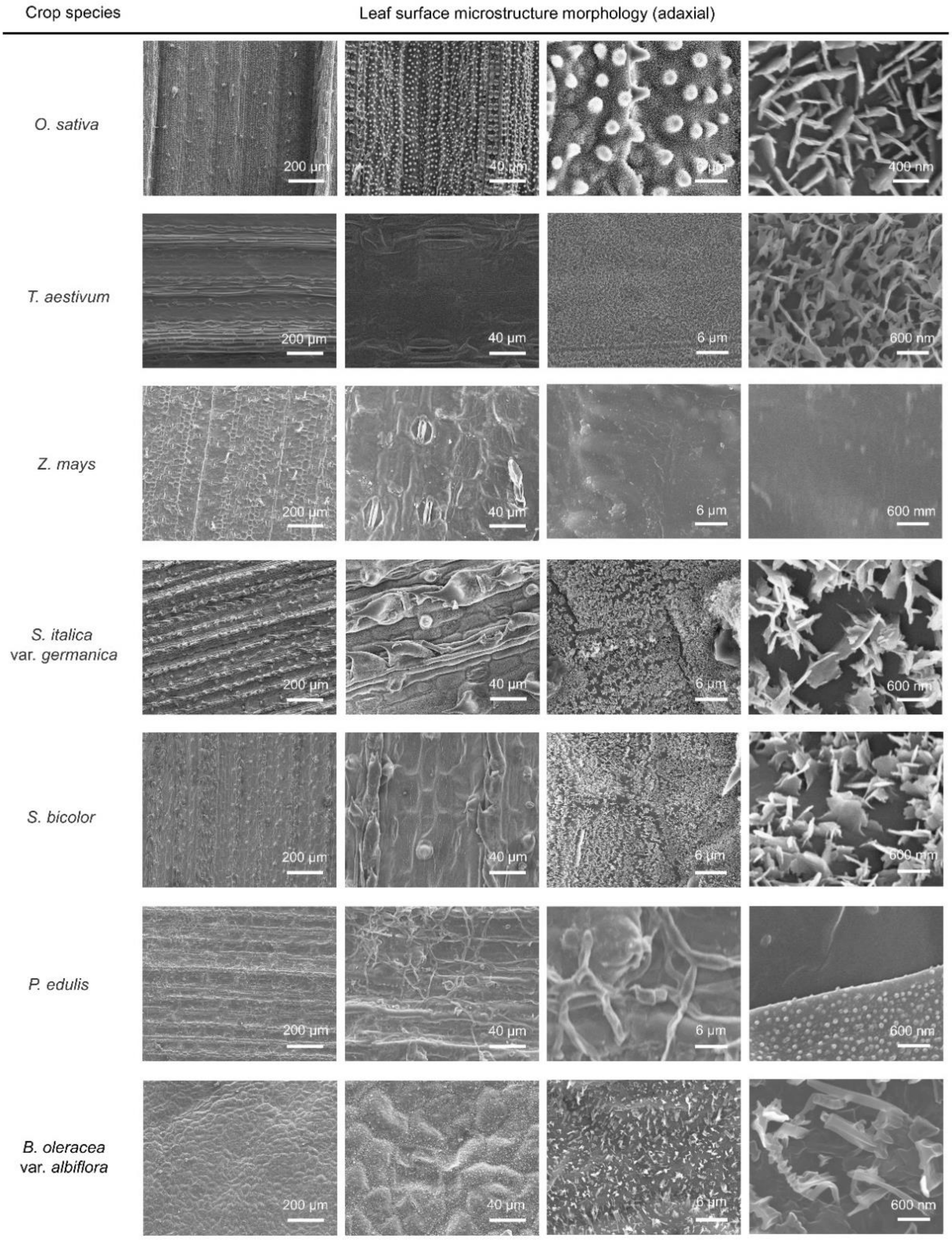

(Continued on the next page)

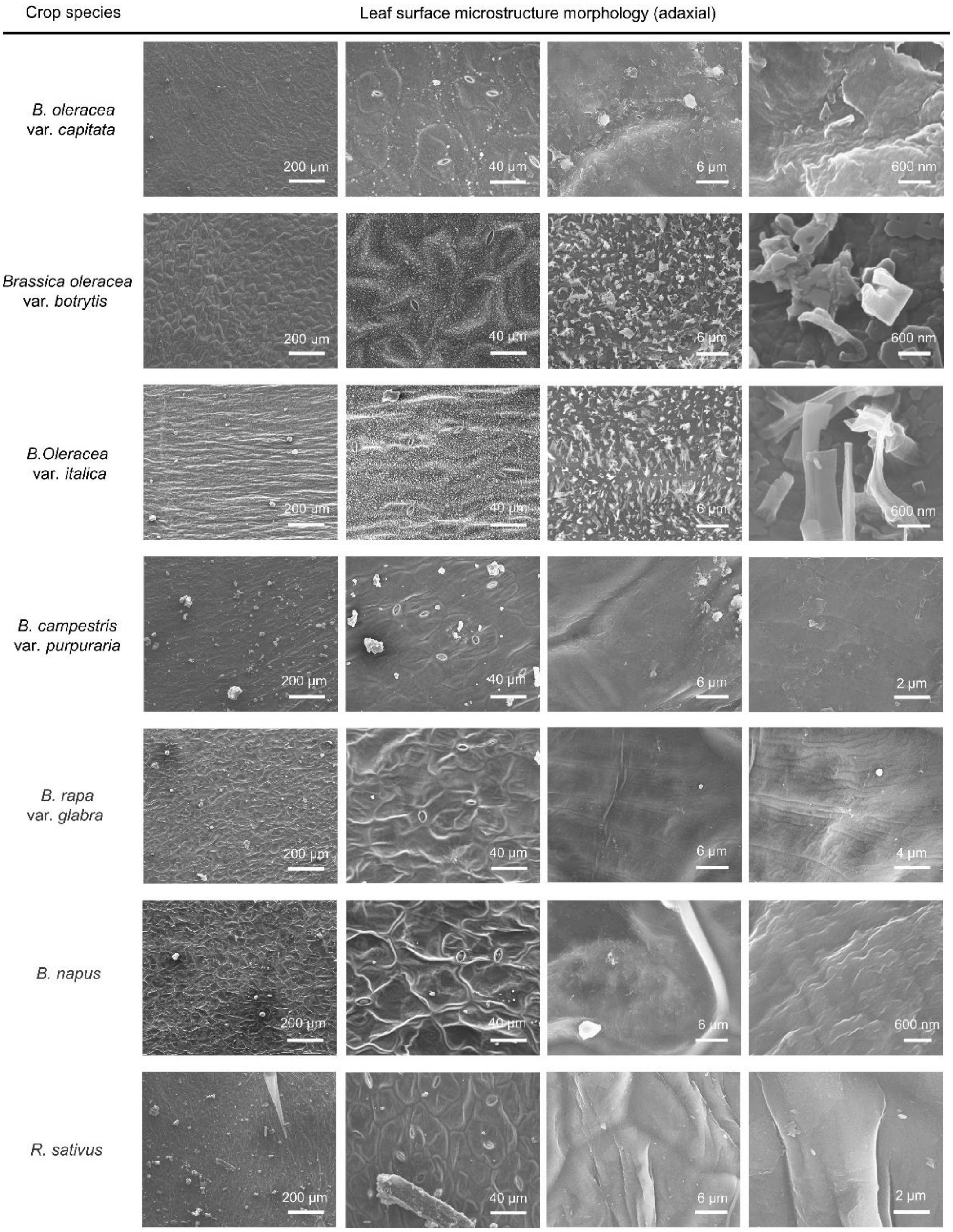

(Continued on the next page)

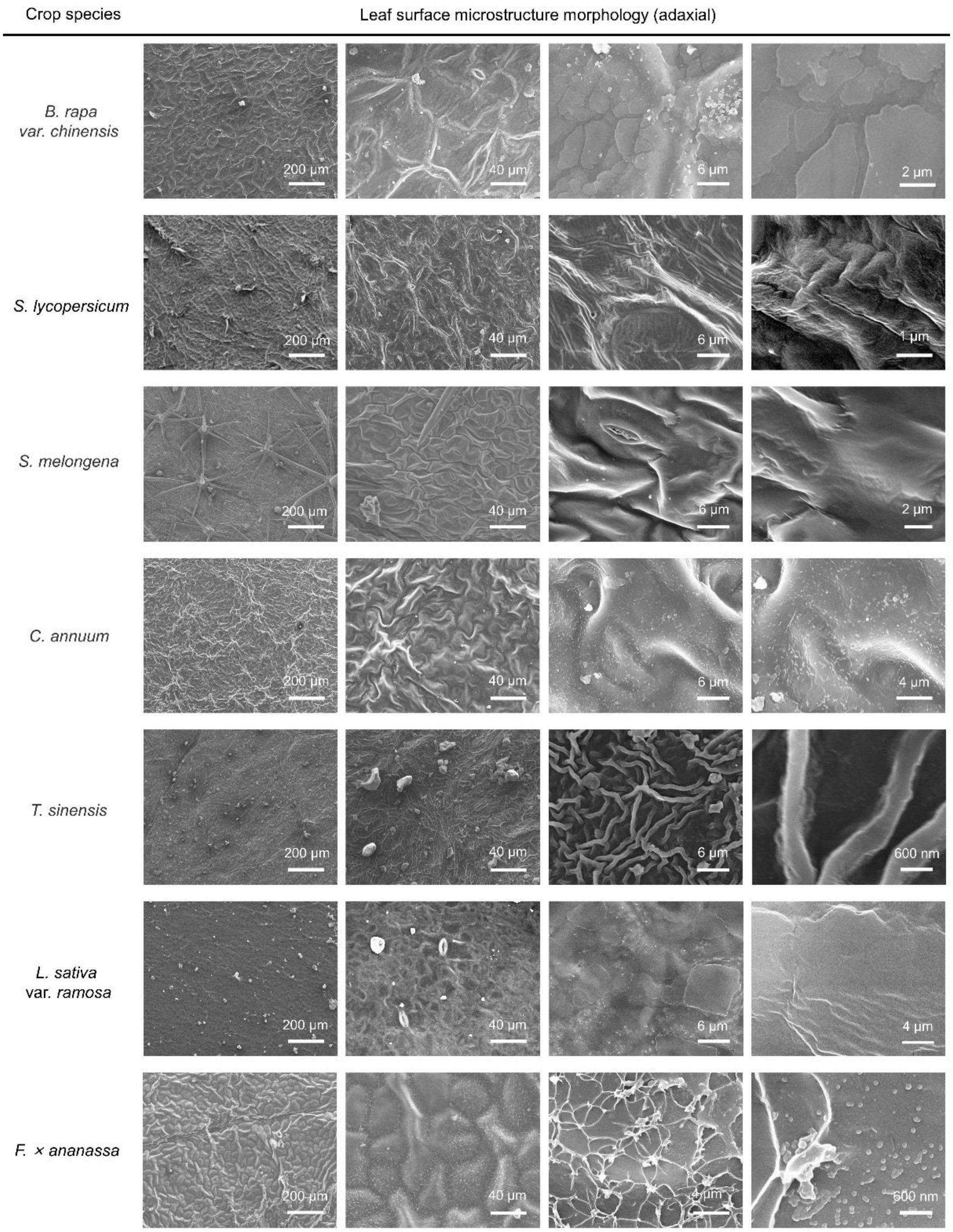

(Continued on the next page)

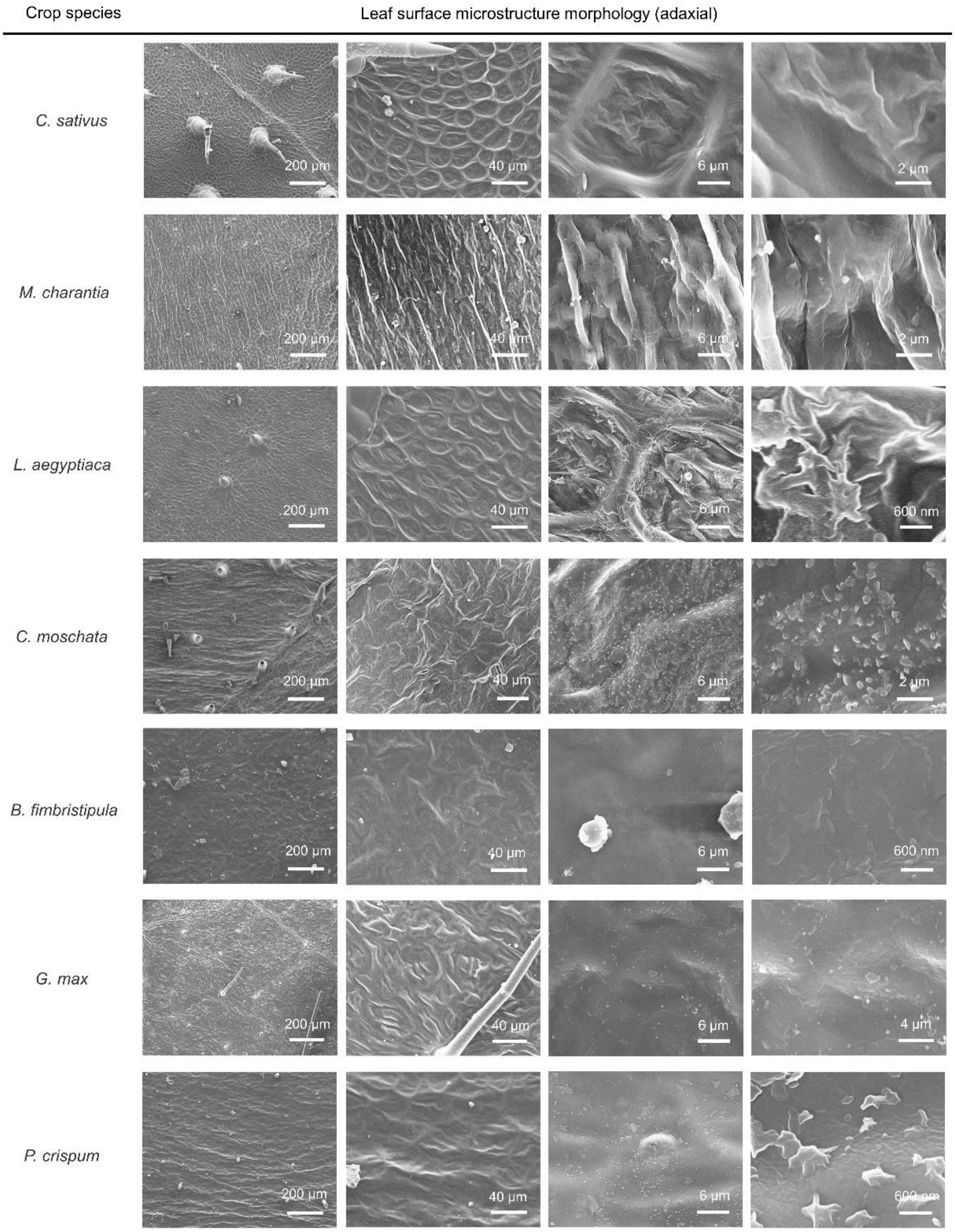

(Continued on the next page)

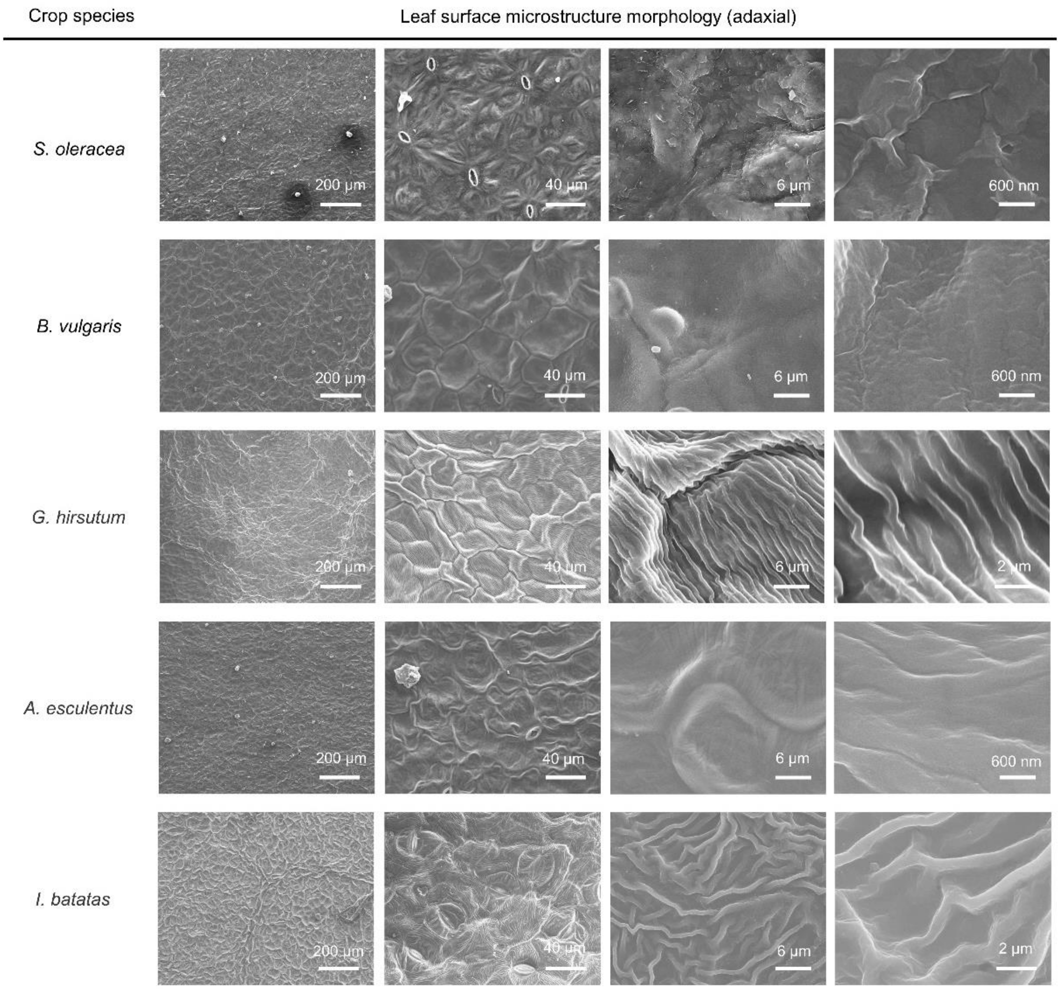

Figure S2. Surface microstructure morphology of crop leaf adaxial surface.

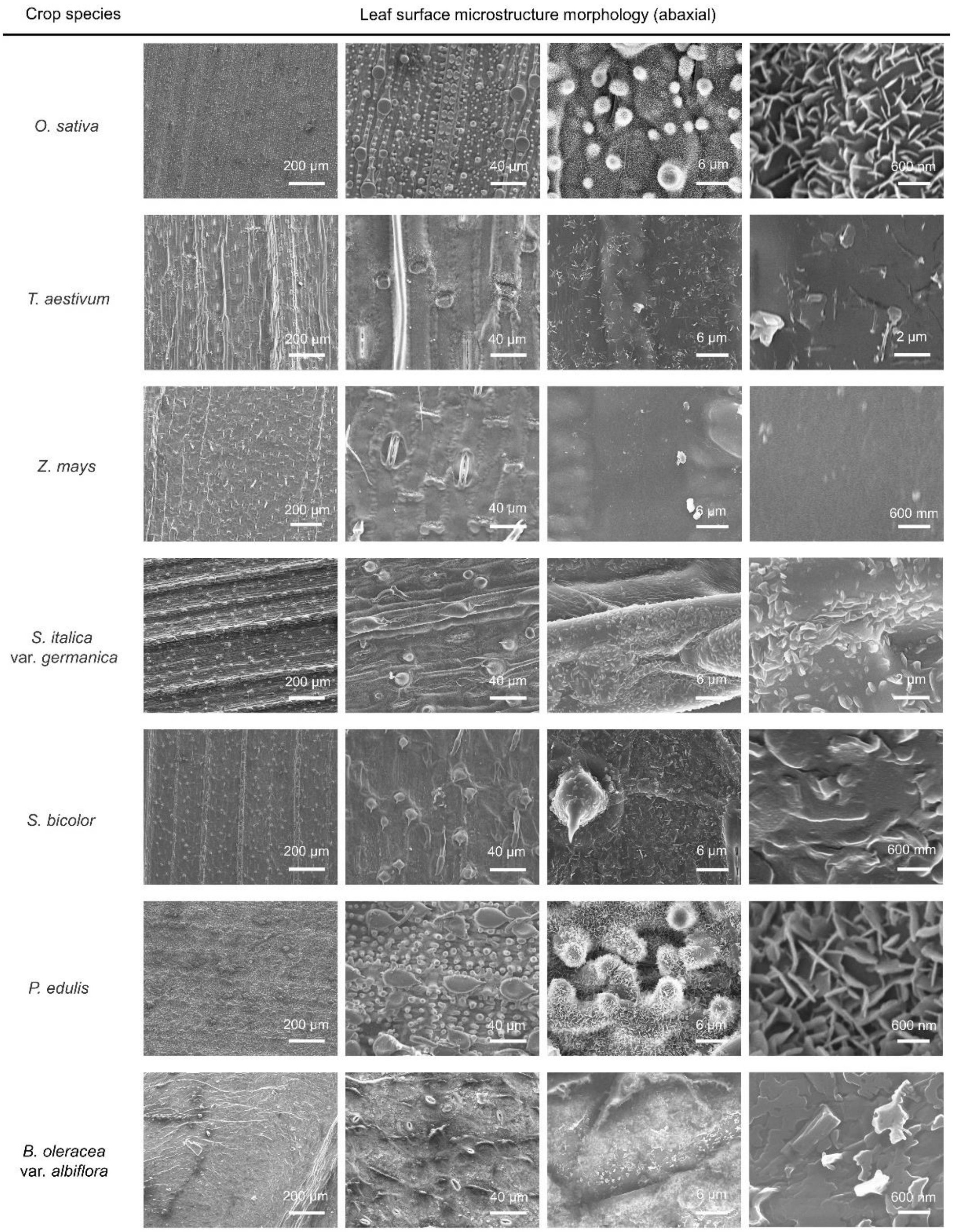

(Continued on the next page)

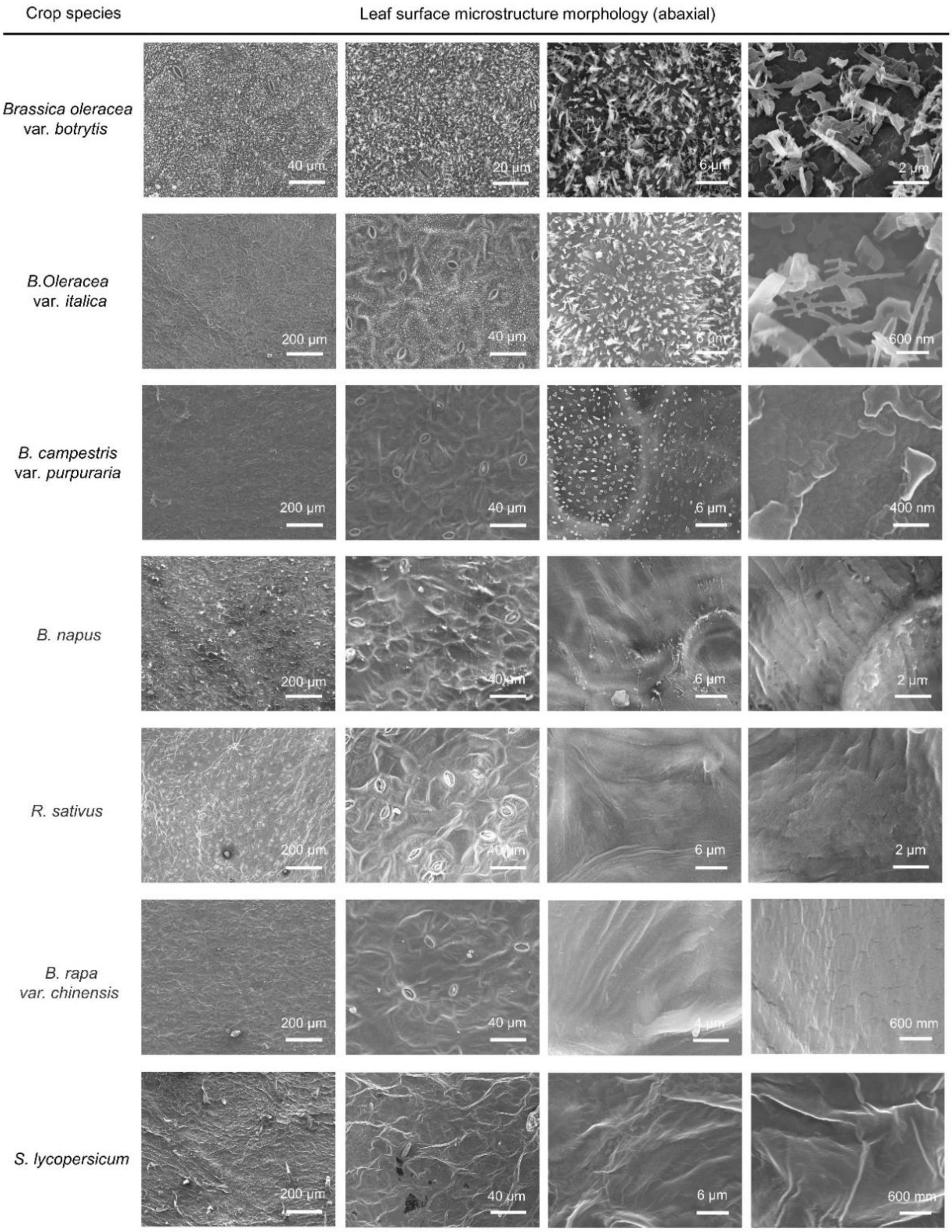

(Continued on the next page)

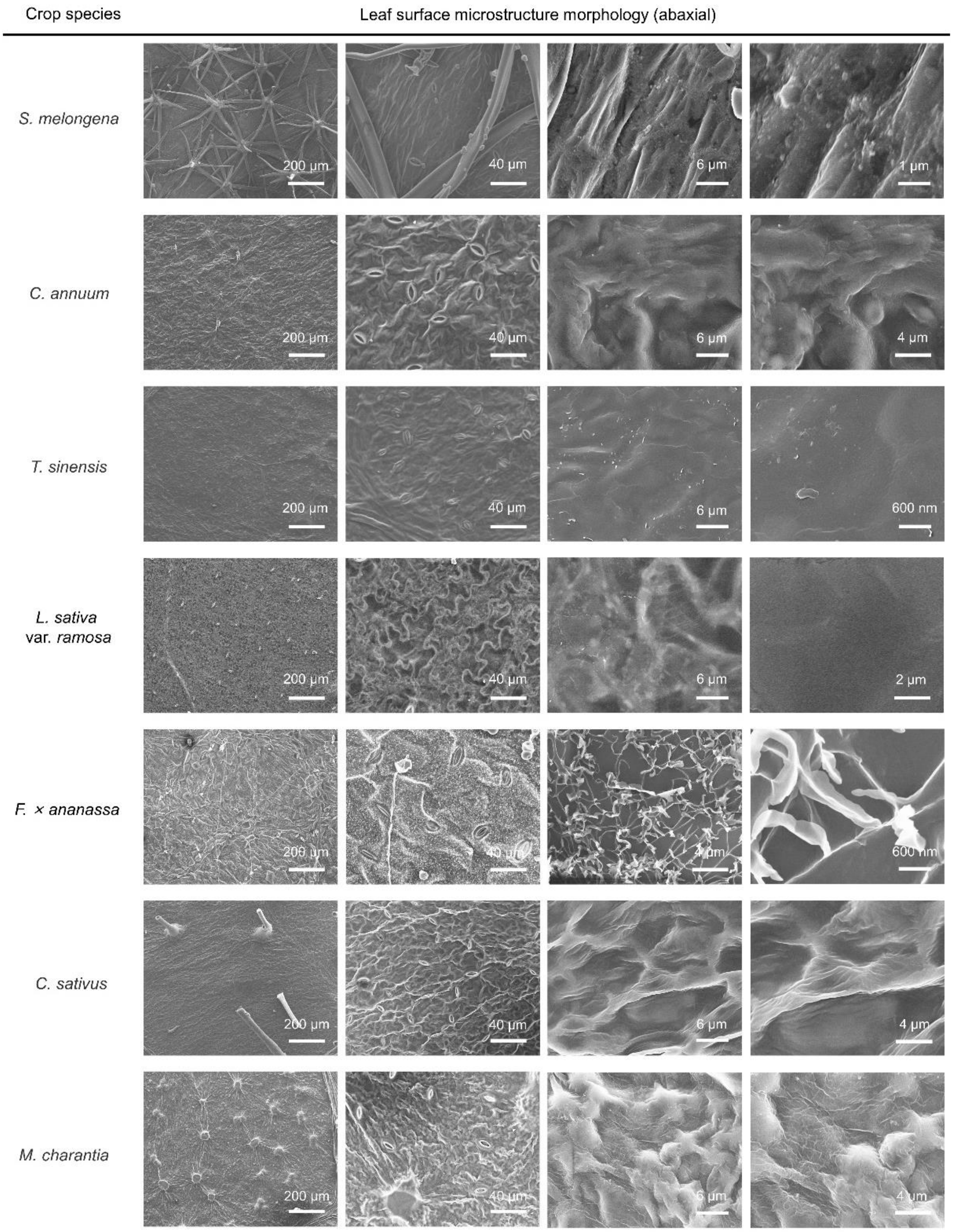

(Continued on the next page)

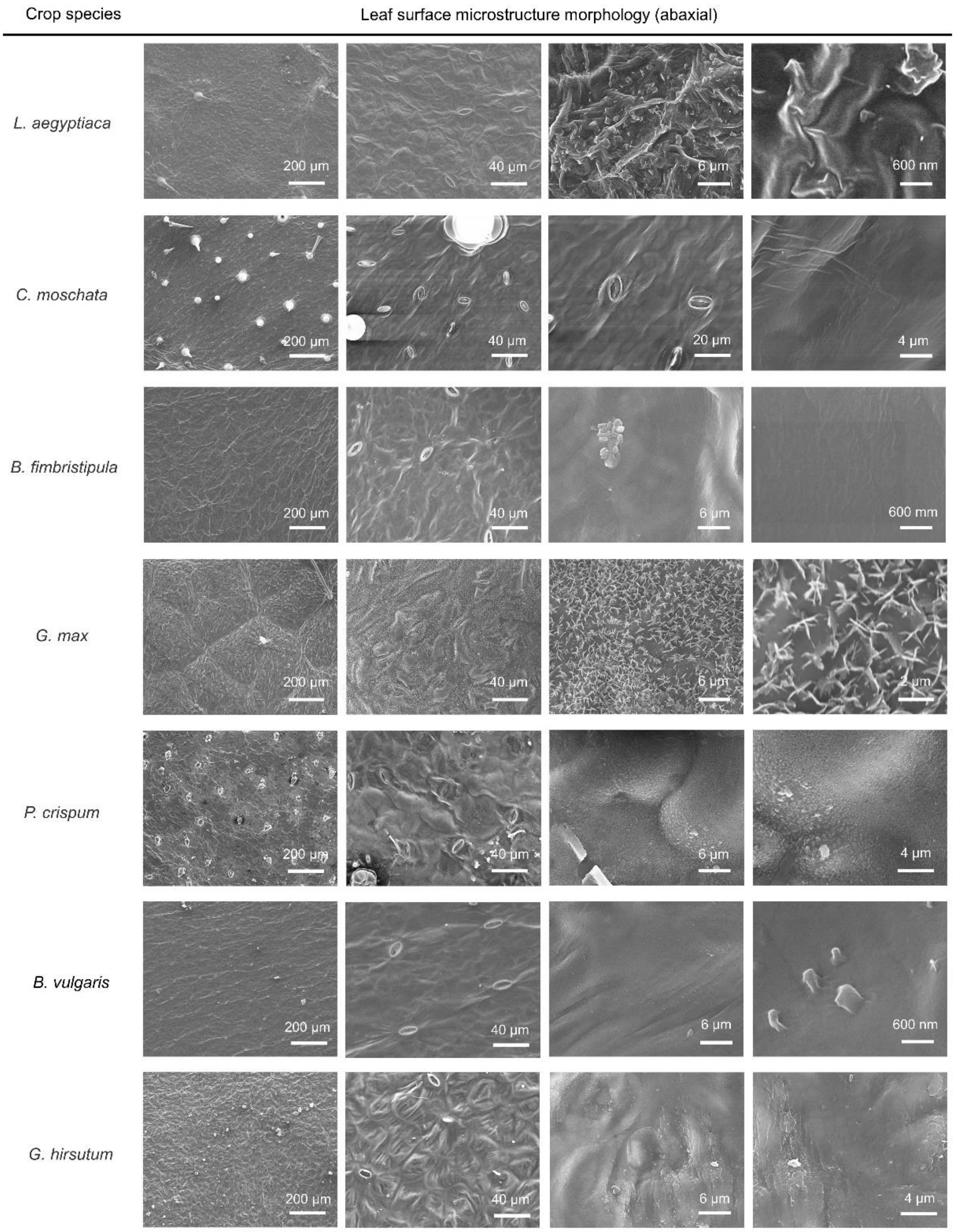

(Continued on the next page)

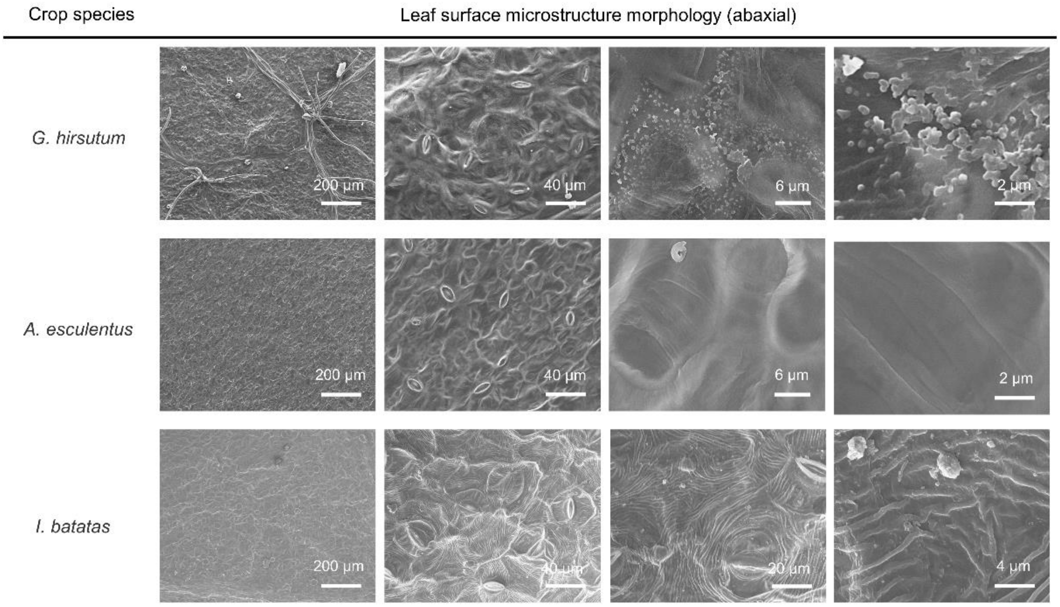

Figure S3. Surface microstructure morphology of crop leaf abaxial surface.

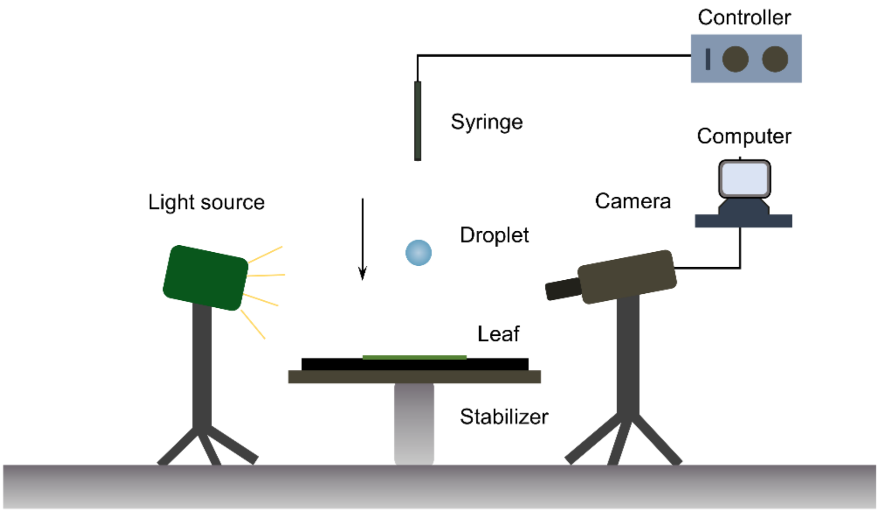

Figure S4. Schematic diagram of the droplet impact experiment by the high-speed camera system.

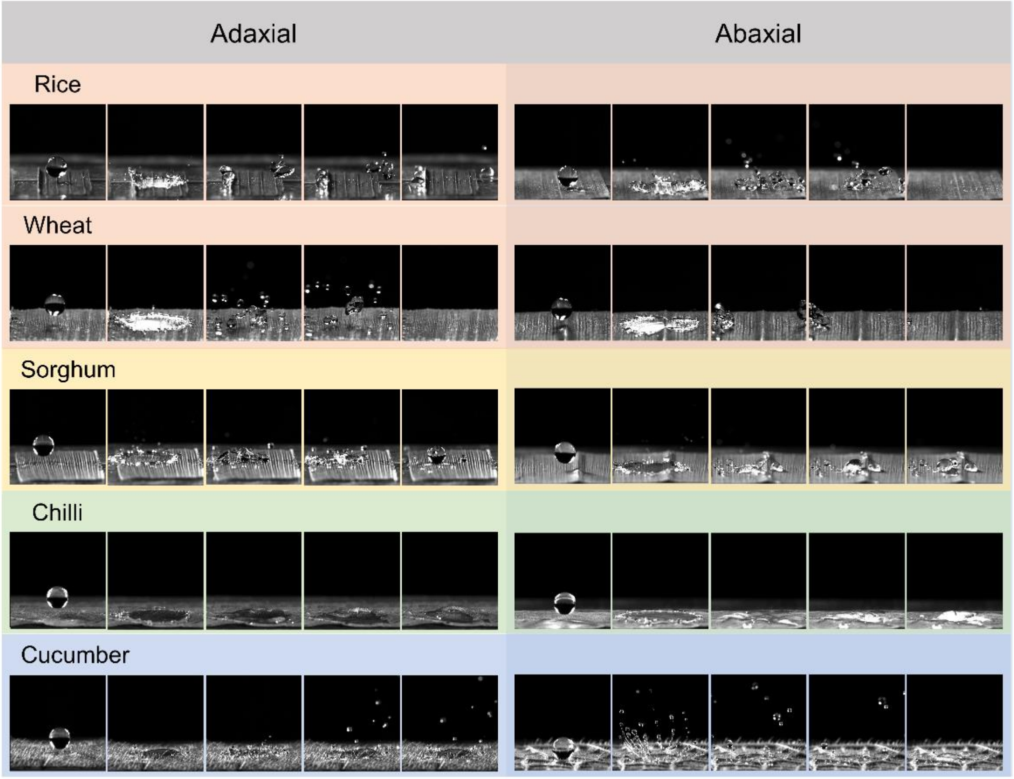

Figure S5. Images of the impact process of several crop leaves with typical wettability.

Surfactant	Molecular structure
SDS	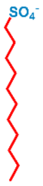
DTAB	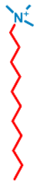
DDAB	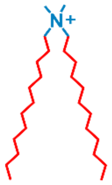
12-3-12	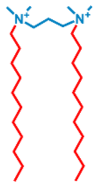
12-3-12-3-12	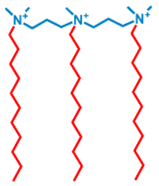
Tx-100	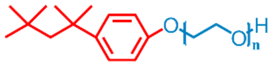
DDAD	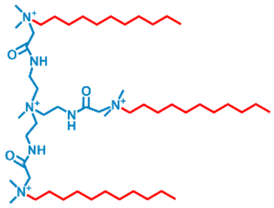

Figure S5. Molecular structures of the surfactants used.

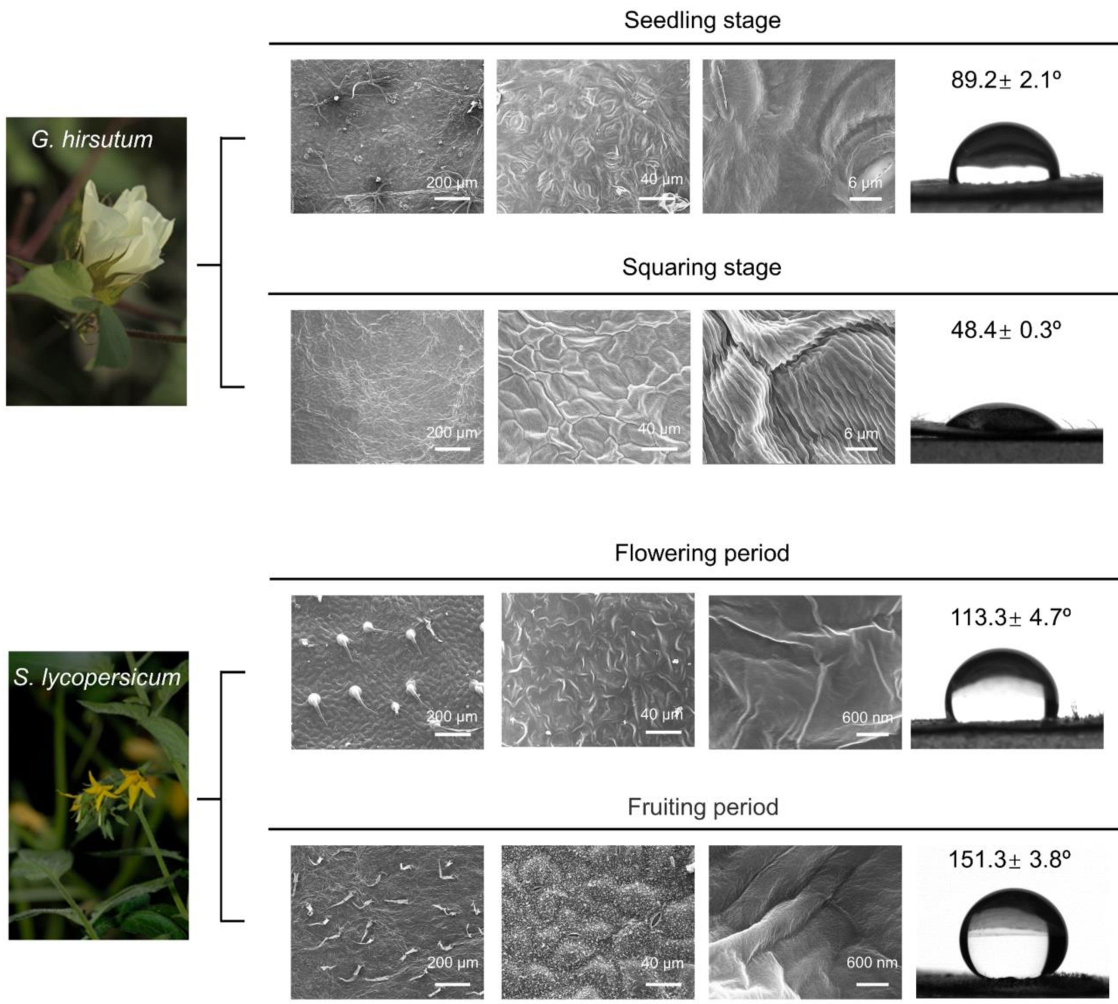

Figure S6. Leaf surface microstructure morphology and corresponding static contact angles of cotton and tomato at different growth stages.

Cotton exhibits distinct leaf microstructures at seedling and squaring stages, with dense micro-ribbed structures gradually stretching out and becoming smooth and sparse, resulting in a shift in static contact angle from 89.2º to 48.4º. Another instance is tomato, with the transition of the growth stage from flowering to fruiting, the waxy leaf layer thickens, the microstructure morphology becomes more complex and multi-layered, closer to wheat, and the static contact angle transforms from 113.3º up to 153.3º.

Table S1. Contact angle (°), advancing angle (°), receding angle (°), and contact angle hysteresis (°) of crop leaves with typical wettability.

Crop species	Contact angle		Advancing angle		Receding angle		Contact angle hysteresis	
	adaxial	abaxial	adaxial	abaxial	adaxial	abaxial	adaxial	abaxial
Rice	158.7 ± 1.4	157.0 ± 1.7	164.0 ± 1.6	162.6 ± 2.7	155.0 ± 1.6	153.2 ± 1.0	9.0 ± 2.3	9.4 ± 2.9
Wheat	152.4 ± 0.5	146.0 ± 2.8	156.7 ± 1.7	151.4 ± 2.2	145.7 ± 2.6	141.5 ± 4.5	11.0 ± 3.1	9.8 ± 5.0
Sorghum	149.3 ± 3.6	133.7 ± 2.9	150.7 ± 2.6	141.4 ± 2.8	139.3 ± 4.9	127.2 ± 3.7	11.4 ± 5.5	14.1 ± 5.1
Galangal	93.5 ± 2.0	116.9 ± 0.7	109.1 ± 3.3	119.9 ± 3.4	15.4 ± 3.5	73.2 ± 1.6	93.7 ± 4.8	46.7 ± 3.4
Chilli	92.4 ± 4.7	91.7 ± 5.7	123.1 ± 2.3	115.4 ± 4.7	54.9 ± 2.8	101.4 ± 2.0	68.1 ± 3.6	13.9 ± 5.1
Cucumber	73.3 ± 6.7	88.9 ± 6.3	108.7 ± 10.3	117.7 ± 5.9	35.0 ± 3.5	62.4 ± 2.2	73.7 ± 10.9	55.3 ± 6.3
